# Effect of number and position of intraocular lens haptics on anterior capsule contraction: a randomized, prospective trial

**DOI:** 10.1186/s12886-018-0742-1

**Published:** 2018-03-20

**Authors:** Mihyun Choi, Marjorie Z. Lazo, Minji Kang, Jeehye Lee, Choun-Ki Joo

**Affiliations:** 1 Department of Ophthalmology, College of Medicine, Seoul St. Mary’s Hospital, The Catholic University of Korea, 222, Banpo-daero, Seocho-gu, Seoul, 06591 Republic of Korea; 2 Catholic Institute for Visual Science, College of Medicine, Seoul St. Mary’s Hospital, The Catholic University of Korea, Seoul, Republic of Korea

**Keywords:** Anterior capsule contraction syndrome, Anterior capsule of the lens, Capsulorhexis, Intraocular lens, Cataract surgery

## Abstract

**Background:**

The present study aimed to evaluate the degree of anterior capsule contraction (capsulorhexis contraction) with three different single-piece, hydrophilic acrylic intraocular lenses (IOLs).

**Methods:**

Patients were prospectively randomized to be implanted with one of three types of IOLs during cataract surgery: the Ophtec Precizon (IOL A), the Lucid Korea Microflex (IOL B), and the Carl Zeiss Asphina (IOL C). One week, 2 weeks, and 6 months after surgery, the area of the anterior capsule opening was measured using digital retro-illumination images after dilation of the pupil. The data were then evaluated using POCOman software.

**Results:**

The study included 236 eyes of 202 patients. The area of the anterior capsule opening reduced by 3.53 ± 3.31 mm (17.06% ± 15.99%) between 1 week and 2 months post-operatively in the IOL A group, by 0.62 ± 1.32 mm (2.87% ± 6.03%) in the IOL B group, and by 1.09 ± 1.53 mm (4.72% ± 6.10%) in the IOL C group. The IOL B group showed minimal anterior capsule contraction 2 months after surgery (*p* < 0.001).

**Conclusions:**

IOLs with a four-plate haptic design (IOL B) showed more anterior capsular stability than those with a two-loop plate haptic (IOL A) or two-plate haptic (IOL C) design. The number and position of haptics in a capsular bag may affect anterior capsule contraction. We assume that supporting the zonules evenly may play a role in anterior capsular stability.

**Trial registration:**

Current Controlled Trials ISRCTN76566080, Retrospectively registered (Date of registration: 14 Feb 2018).

## Background

Anterior and posterior capsular opacification are still major complications of cataract surgery that occur due to the proliferation, migration, and differentiation of residual lens epithelial cells (LECs) [1, 2]. Contact with the intraocular lens (IOL) optic causes the LECs of the anterior lens capsule to undergo fibrosis, resulting in anterior capsule opacification (ACO) [1, 3]. This may in turn lead to contraction or retraction of the anterior capsule, and ultimately to a reduction in the free optic zone. Furthermore, anterior capsule contraction (ACC) may cause decentration of the IOL optic, as well as tilt of the IOL, and zonular stretching may lead to zonular rupture and subsequent dislocation of the IOL/capsular bag posteriorly [4–6].

The prevalence of posterior capsule opacification (PCO) has decreased due to technological and surgical improvements. Specifically, neodymium:yttrium-aluminum-garnet (Nd:YAG) laser capsulotomy is an effective PCO treatment option [7]. However, treatment can lead to further complications, including cystoid macular edema, IOL subluxation and damage, elevation of intraocular pressure, and retinal detachment [8, 9]. For these reasons, it remains important to prevent PCO. To do so, IOL manufacturers continuously modify IOL designs and materials, and ophthalmic surgeons refine their surgical techniques to minimize the ACO and PCO risk.

Previous studies have reported that capsular fibrosis occurs 90–180 days after implantation [10], and the degree of ACC has been associated with many predictors, including individual, pathological, and surgical factors [11–13]. For instance, Hayashi et al. reported that the mean percentage reduction in the anterior capsule opening area was significantly greater in eyes with a silicone optic IOL than in eyes with an acrylic optic IOL. The same authors reported that the one-piece acrylic IOL appeared to withstand substantial post-operative capsular shrinkage, and that its optic and haptic design was not associated with ACC [3, 14]. In another study, Hayashi et al. observed no significant difference in ACC occurrence between acrylic IOLs with round-edge optics and those with sharp-edge optics [15]. Conversely, Sacu et al. reported that neither the material nor the haptic design of hydrophobic IOLs affected the occurrence of ACO or ACC [16]. A 2010 study found that greater ACC occurs after hydrophilic IOL implantation than after hydrophobic IOL implantation [17]. However, these studies only evaluated the material and edge type of the optics and the shape of the haptics, not the number and position of the haptics within the capsular bag. ACC occurs when the centripetal force of the anterior capsule opening margin (fibrotic change) differs from the centrifugal force of the capsular zonule [18, 19]. In this regard, to investigate whether the number and position of IOL haptics affected ACC, the present study evaluated the capsulorhexis aperture after implantation of three differently designed IOLs. The study also evaluated the rate of PCO for each IOL.

## Methods

### Patient recruitment, randomization, and intraocular lenses

This was a prospective, randomized study of patients who were to undergo cataract surgery at Seoul St. Mary’s Hospital, Seoul, South Korea, between August 2016 and December 2016. Two-hundred thirty-six eyes of 202 patients were included. The study protocol followed the guidelines of the Declaration of Helsinki. Potential complications were explained to the patients in detail, and written informed consent was obtained before the study began. The inclusion criteria were (1) age over 55 years and under 75, (2) presence of age-related cataract, (3) axial length within the normal range (22–25.5 mm), and (4) dilated pupil larger than 8.0 mm in diameter. The exclusion criteria were histories of (1) ocular disease, (2) intraocular surgery, (3) laser treatment, (4) glaucoma, and (5) severe retinal pathology. Each patient underwent a complete ophthalmologic evaluation before their planned cataract surgery. During this evaluation, the status of the zonule was assessed. Specifically, 2.5% phenylephrine was instilled into the eyes of the patients; 30 min later, slit-lamp biomicroscopy was performed, with attention to lenticular centration or malposition, iris transillumination defects, pseudoexfoliation material on the anterior lens capsule or pupil margin, and phacodonesis (looseness that manifests as jiggling movements on the slit lamp). Patients with the following conditions known to affect ACC were also excluded: (1) retinitis pigmentosa, (2) diabetic retinopathy, (3) myotonic dystrophy, (4) uveitis, (5) old age (over 75 years), and (6) pseudoexfoliation syndrome [20].

Before the study began, a simple randomization was performed using Excel™ software (Version 2010; Microsoft). One of the following three randomly assigned IOLs was implanted during each patient’s cataract surgery: Precizon IOL (OPHTEC; IOL A), Microflex IOL (Lucid Korea Inc.; IOL B), and CT Asphina 509 M IOL (Carl Zeiss; IOL C).

These three IOLs have different haptic designs, but all are made of acrylic material and are single-piece lenses (Table 1). All three have a hydrophilic acrylic characteristic, but the Asphina (IOL C) has hydrophobic surface properties. Each IOL has a different number and position of haptics. The Precizon (IOL A) has a two-loop plate haptic at a 180° interval around the optic, while the Microflex (IOL B) has a four-loop plate haptic at 96° and 84° intervals around the optic. The Asphina has a plate-shaped haptic (IOL C) at a 180° interval around the optic.

**Table 1:** Characteristics of the three types of acrylic intraocular lenses

	Precizon (IOL A)	Microflex (IOL B)	Asphina (IOL C)
Optic material	hydrophilic acrylic	hydrophilic acrylic	Hydrophilic acrylic (25%) with hydrophobic surface properties
Haptic material	hydrophilic acrylic	hydrophilic acrylic	Hydrophilic acrylic (25%) with hydrophobic surface properties
Optic diameter	6.0 mm	6.0 mm	6.0 mm
Overall length	12.5 mm	10.5 mm	11.0 mm
Haptic design	2 Plate loop design;sharp edged	4 Plate loop design; round-edged	2 Plate design(no loop);sharpedged
Haptic angulation	0°	5°	0°
Optic type	Biconvex	Biconvex	Biconvex
Sphericity	Aspheric	Spheric	Aspheric

### Surgical technique

The same experienced surgeon (C.K.J.) performed all cataract surgeries using a standard procedure. A 2.2-mm clear corneal incision was made, and 1.4% sodium hyaluronate (Healon GV) was injected into the anterior chamber to maintain the chamber’s stability. To allow the area of the anterior capsule opening to be compared between groups after surgery, surgeons had to perform a continuous curvilinear capsulorhexis (CCC) with a specific size during each surgery. For this purpose, the Open Ring-shaped Guide for CCC [21] (ORGC; Lucid Korea, Inc.) was used. This is a ring-shaped ruler with an open 10° arc that guides targeted CCC size. The surgeon selected the size of the ORGC based on the IOL to be used. Specifically, an ORGC diameter of 5.2 mm was used with the Precizon, while a diameter of 5.4 mm was used with the Microflex and Asphina. The ORGC serves as a guideline for CCC, helping the surgeon to make an exact, symmetrical, 360-degree capsulorhexis–IOL overlap. The CCC was carefully performed using capsulorhexis forceps following the internal border of the guide. After thorough hydrodissection, phacoemulsification of the nucleus and aspiration of the residual cortex were performed using an Infiniti™ gravity-fluidics torsional phacoemulsification machine (Alcon Laboratories, Inc.). The folded IOLs were implanted in the bag with an injector. After IOL implantation, the ophthalmic viscoelastic device (OVD) was carefully removed from the anterior chamber and capsular bag by coaxial irrigation/aspiration (I/A). Specifically, OVD aspiration from the bag was facilitated by tilting the IOL slightly and positioning the I/A tip behind the IOL optic. Anterior capsule polishing, which can affect the residual LECs of the anterior capsule, *was not performed* [22]. There were no surgical complications leading to patient exclusion. Post-operative treatment consisted of prednisolone acetate (1.0%; Pred Forte®) and moxifloxacin (Vigamox®) eye drops 4 times a day for 1 month. Follow-up examinations were performed at 1 week (± 2 days), 2 months (± 14 days), and 6 months (± 1 month).

### Assessment of anterior capsule opening size and posterior capsule opacification using standardized retro-illumination photography

At each follow-up visit, LogMAR visual acuity (corrected distance visual acuity [CDVA]) and refraction were tested, and a slit-lamp examination was performed. Patients received 2.5% phenylephrine at least 30 min before the digital retro-illumination images were taken.

**Fig. 1:**
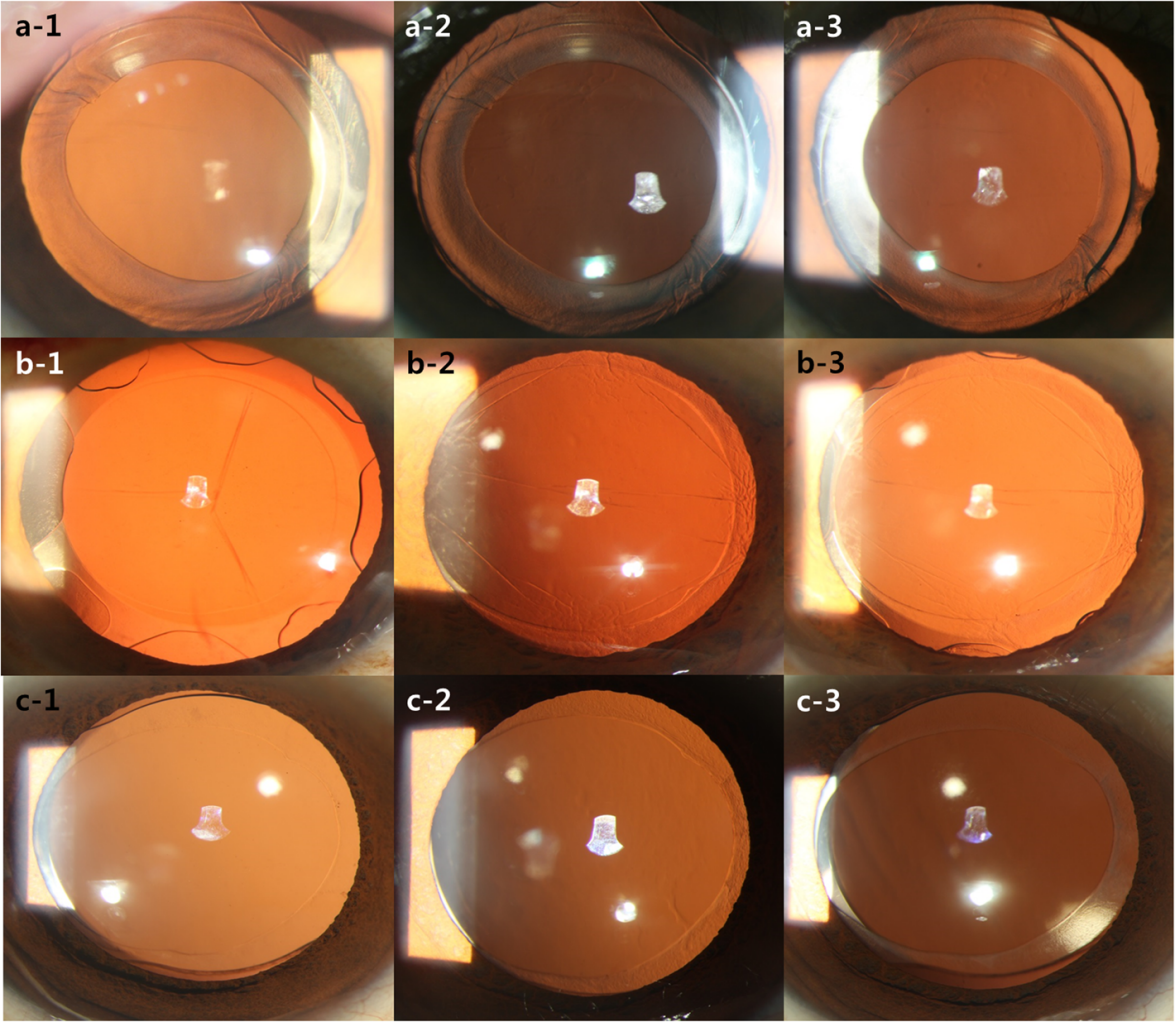
Digital retro-illumination images of implanted (**a**) two-loop plate-haptic IOL, (**b**) 4-loop plate-haptic IOL, (**c**) plate-haptic IOL, at 1 week (1), 2 months (2) and 6 months (3) after surgery. (IOL = intraocular lens)

The digital retro-illumination images were taken from the anterior and posterior capsule at each visit using a digital camera (Sony HDR-CX300) mounted on a modified Zeiss 30 slit lamp with an external light and flash light source [7]. The operator measured the area of the anterior capsule opening by analyzing the image of digital retro-illumination using POCOman software [23]; Fig. 1). PCO area and severity were also measured in the retro-illumination images using POCOman software. All measurements were repeated three times and performed by a single experienced technician. The average measurement was used in the statistical analysis. POCOman was introduced in 2004 as an objective and repeatable method that uses retro-illumination images to quantify PCO area and severity, as well as the area of the anterior capsule opening. Since the diameters of IOL optics were the same in each group, the actual area of the anterior capsule opening was calculated in proportion to the optic size. The POCOman software measures the area of the anterior capsule opening by the operator setting the opening area scale; this is done subjectively. The software can also analyze PCO by texture analysis to derive the percentage area and severity of PCO in a semi-objective manner by allowing the user to define the PCO within the automatically calculated image.

### Statistical analysis

Statistical analysis was performed using IBM SPSS Statistics ver. 24.0 (IBM SPSS Statistics for Windows, Version 24.0., Armonk, NY, USA). Paired t-tests and Mann–Whitney U tests were used for pairwise comparisons. The Kruskal–Wallis test and one-way ANOVA were used to compare the three IOLs. All data were expressed as mean ± standard deviation. A *p*-value of less than 0.05 was considered statistically significant.

## Results

Two hundred thirty-six eyes of 202 patients were included in the study. The mean age of the patients at surgery was 65.43 years ±9.41 (SD) (range 55–75 years). One-hundred thirty-eight (58.5%) were women and 98 (41.5%) were men. At the 6-month follow-up examination, 186 patients (92.1%) were examined. No adverse event occurred during this study.

Seventy-six eyes (67 patients) were implanted with the IOL A, 78 eyes (66 patients) with the IOL B, and 82 eyes (69 patients) with the IOL C. The average patient age in each group was 64.79 ± 10.22 years, 66.61 ± 9.2 years, and 64.40 ± 10.29 years, respectively (*p* = 0.533). The characteristics of the patients in the three groups are presented in Table 2. No significant differences were found regarding male-to-female ratio (*p* = 0.106), ratio of left to right eyes (*p* = 0.301), or axial length (*p* = 0.438) between the groups. Patients with diabetic retinopathy were excluded. However, those with diabetes alone, which is also known to affect ACC, were not excluded. Regardless, there was no significant difference in the proportion of diabetic patients among the three groups (*p* = 0.643; Table 2). The target diopter was calculated using the SRK-T formula as the predicted refractive error after cataract surgery. The IOL C group showed a more myopic target than the IOL A and IOL B groups (− 0.66 ± 0.91, − 0.61 ± 0.84, and − 1.01 ± 1.09, respectively; *p* = 0.016). However, there was no significant difference in the diopter of the implanted IOL (*p* = 0.208; Table 2).

**Table 2:** Patient Characteristics

	Precizon (IOL A)	Microflex (IOL B)	Asphina (IOL C)	*P-value* ^a^
Eyes (*n*)	76	78	82	
Mean age(yr)	64.79 ± 10.22	66.61 ± 9.2	64.40 ± 10.29	0.533
Male/Female	28/48	34/44	36/46	0.106
DM (*n*)	5	7	7	0.643
OD/OS	33/42	36/42	48/34	0.301
Axial length (mm)	23.46 ± 3.11	23.92 ± 2.81	23.22 ± 2.75	0.438
CDVA before surgery(log MAR)	0.2 ± 0.16	0.35 ± 0.24	0.28 ± 0.31	0.501
Diopter (D) (range)	19.73 ± 3.53 (7.0 ~ 25.5)	20.26 ± 1.99 (16.5 ~ 25.0)	18.7 ± 3.51 (8.0~ 24.5)	0.208
Target (D) (range)	−0.66 ± 0.91 (− 2.78~ 0.04)	−0.61 ± 0.84 (− 2.99~ 0.06)	− 1.01 ± 1.09 (− 2.98 ~ 0.0)	0.016

^a^ Kruskal Wallis test

### Visual acuity

The pre-operative mean CDVA values were 0.25 ± 0.22 in the IOL A group, 0.34 ± 0.37 in the IOL B group, and 0.29 ± 0.33 in the IOL C group (*p* = 0.17). At the 2-month follow-up, the values were 0.05 ± 0.1, 0.05 ± 0.08, and 0.05 ± 0.14 (*p* = 0.36), respectively. At the 6-month follow-up, they were 0.04 ± 0.08, 0.06 ± 0.08, and 0.07 ± 0.11 (*p* = 0.19), respectively. There was no significant difference in CDVA among the three groups either before or after surgery.

### Anterior capsule opening size

**Fig. 2:**
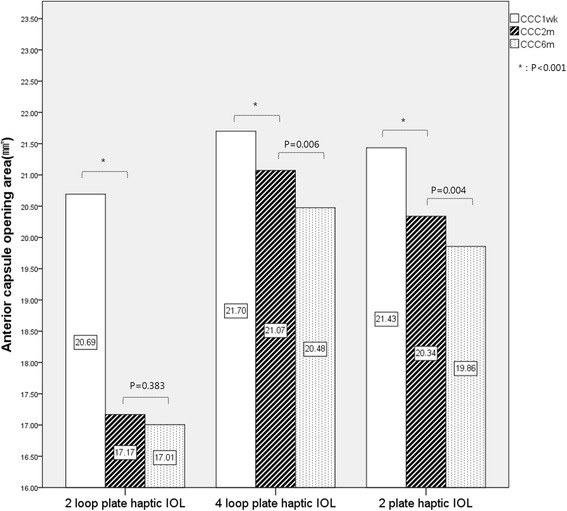
Mean area of the anterior capsule opening in the three groups (mm^2^)

**Fig. 3:**
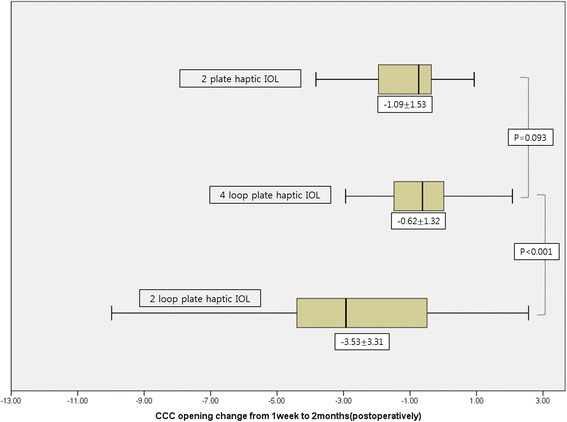
Change in anterior capsule opening size from 1 week to 2 months post-operatively (mm^2^)

Table 3 summarizes the mean (± SD) area of the anterior capsule opening in each of the three groups. The mean areas of the anterior capsule opening at 1 week, 2 weeks, and 6 months after surgery were 20.69 ± 1.50 mm^2^, 17.17 ± 3.23 mm^2^, and 17.01 ± 3.11 mm^2^, respectively, in the IOL A group; 21.70 ± 0.86 mm^2^, 21.07 ± 1.50 mm^2^, and 20.48 ± 1.09 mm^2^, respectively, in the IOL B group; and 21.43 ± 1.09 mm^2^, 20.34 ± 1.60 mm^2^, and 19.86 ± 1.58 mm^2^, respectively, in IOL C group. The area of the anterior capsule opening decreased in all three groups from 1 week to 2 months after surgery, and from 2 months to 6 months after surgery. However, the reduction was greatest in the first 2 months after surgery (Fig. 2). The area of the anterior capsule opening after 1 week was smallest in the IOL A group, but no significant difference was observed among the three groups in this regard (*p* = 0.068; Table 3). At 2 and 6 months after surgery, the area of the anterior capsule opening was significantly smaller in eyes with an IOL A than in eyes with an IOL B or IOL C (*p* < 0.001). The reduction in the area of the anterior capsule opening between 1 week and 2 months after surgery was 3.53 ± 3.31 mm^2^ (17.06% ± 15.99%) in the IOL A group, 0.62 ± 1.32 mm^2^ (2.87% ± 6.03%) in the IOL B group, and 1.09 ± 1.53 mm^2^ (4.72% ± 6.10%) in the IOL C group (Fig. 3). Thus, the IOL A showed the lowest anterior capsular stability after surgery, while the IOL B showed the greatest anterior capsular stability (*p* < 0.001; Table 3). In addition, with regards to the ACC that occurred between 1 week and 2 months after surgery, the IOL B showed a smaller reduction in the area of the anterior capsular opening than did the IOL C, though this was not significant (*p* = 0.093; Fig. 3).

**Table 3:** Mean Area of the Anterior Capsule Opening and Change after Cataract Surgery at Postoperative Follow Up (mm2)

Follow up	1wk	2 m		6 m	
	CCC size	CCC size	Change from 1 week(percentage)	CCC size	Change from 2 months(percentage)
Precizon (IOL A)	20.69 ± 1.50	17.17 ± 3.23	−3.53 ± 3.31 (17.06 ± 15.99%)	17.01 ± 3.11	− 0.16 ± 1.31 (0.64 ± 6.21%)
Microflex (IOL B)	21.70 ± 0.86	21.07 ± 1.50	− 0.62 ± 1.32 (2.87 ± 6.03%)	20.48 ± 1.09	− 0.60 ± 1.51 (2.47 ± 6.91%)
Asphina (IOL C)	21.43 ± 1.09	20.34 ± 1.60	−1.09 ± 1.53 (4.72 ± 6.10%)	19.86 ± 1.58	−0.48 ± 1.19 (2.12 ± 5.25%)
*P-value*†	0.068	< 0.001	< 0.001	< 0.001	0.170

†one-way ANOVA

### Posterior capsule opacification

Table 4 shows the calculated values for PCO in the three groups using the POCOman software. The mean PCO scores at 1 week, 2 months, and 6 months post-operatively were 6.51 ± 3.34, 11.09 ± 6.81, and 13.75 ± 7.49, respectively in the IOL A group; 6.16 ± 3.87, 27.23 ± 14.90, and 43.26 ± 12.79, respectively, in the IOL B group; and 6.46 ± 5.69, 15.56 ± 8.18, and 16.98 ± 5.48, respectively, in the IOL C group. There was no difference among the three groups 1 week after surgery. However, at 2 and 6 months after surgery, PCO was more pronounced in the IOL B group (*p* < 0.001).

**Table 4:** Mean PCO score and grade at Postoperative Follow Up

Follow up	1wk		2 m		6 m	
	Score	Grade	Score	Grade	Score	Grade
Precizon (IOL A)	6.51 ± 3.34	0.06 ± 0.03	11.09 ± 6.81	0.12 ± 0.09	13.75 ± 7.49	0.15 ± 0.10
Microflex (IOL B)	6.16 ± 3.87	0.06 ± 0.04	27.23 ± 14.90	0.36 ± 0.25	43.26 ± 12.79	0.64 ± 0.32
Asphina (IOL C)	6.46 ± 5.69	0.06 ± 0.06	15.56 ± 8.18	0.16 ± 0.10	16.98 ± 5.48	0.19 ± 0.06
*P-value*	0.409	0.362	< 0.001	< 0.001	< 0.001	< 0.001

### Change in refractive error

Table 5 shows the spherical equivalent (SE) value in each group at the post-operative follow up. As shown in Table 2, the pre-operative refractive targets differed among the three groups, and the IOL C group had a more myopic target. The mean changes in SE values between 1 week and 6 months after surgery were 0.18 ± 0.49 in the IOL A group, − 0.01 ± 0.84 in the IOL B group, and 0.12 ± 0.47 in the IOL C group (*p* = 0.046). The refractive outcome was most stable in the IOL B group. The IOL A and IOL B groups showed a hyperopic shift, and there was no significant difference between two groups (*p* = 0.872).

**Table 5:** Mean Refractive Outcomes (SE) at Postoperative Follow Up

Follow up	1wk	2 m	6 m	Refractive change (1 week to 6 months)
Precizon (IOL A)	−0.70 ± 1.02	−0.49 ± 1.03	−0.50 ± 1.11	0.18 ± 0.49
Microflex (IOL B)	−0.52 ± 1.01	−0.49 ± 1.06	−0.52 ± 0.98	−0.01 ± 0.84
Asphina (IOL C)	−0.80 ± 1.27	−0.73 ± 1.34	−0.65 ± 1.28	0.12 ± 0.47
*P-value*				0.046

*SE* spherical equivalent

## Discussion

The anterior capsule opening decreases in area when LECs remaining on the IOL surface proliferate and undergo fibrous metaplasia upon contacting the IOL [24]. The imbalance between the centripetal force exerted by the LECs (fibrous metaplasia) and the centrifugal force exerted by the zonule induces ACC [18, 19]. Several reports have revealed that ACC occurs more frequently when using silicone IOLs or hydrogel optic IOLs, and that plate-haptic IOLs or thin optic IOLs cause less capsule dilation of the centrifugal haptics [3, 11, 12, 17, 25]. The association between silicone or hydrogel optics and ACC may be due to a weaker adhesion of the optic materials to the lens capsule. Weak adhesion may allow space for active proliferation of LECs and synthesis of extracellular matrix. In contrast, because acrylic optics adhere firmly to the capsule, and remnant LECs are only minimally exposed to various cytokines in the aqueous humor, fibrosis and contraction of the anterior capsule would be reduced when using such IOLs [3, 26, 27]. With specific regard to acrylic IOLs, hydrophilic varieties have shown significantly more frequent ACC than hydrophobic varieties [17], perhaps because hydrophobic IOL material prevents attachment of migrating epithelial cells on the optic and haptic surfaces. Other studies have reported that there is no difference between round- and sharp-edge optic design in terms of ACC prevalence [15, 16]. In summary, studies to date have indicated that optic surface contact with the anterior capsule, the convexity of the optic, and the edges of the optic and haptic, may play a role in LEC growth and anterior capsule contracture.

In the present study, we focused on the number and position of the haptics in the circular zonule, because ACO may be caused by LEC growth, whereas ACC is accelerated by the difference between the centripetal force of the anterior capsule opening margin (fibrotic force) and the centrifugal force of the capsular zonule [18, 19]. Some studies have suggested that four-haptic IOLs confer a large surface that contacts the posterior capsule, as well as more accurate IOL fixation within the capsular bag, leading to constant tension on the zonular fibers [28]. However, later studies reported that the number and design of haptics were not strongly associated with ACC [27]. In the present study, we noticed that it is not only the number of haptics that matters in ACC, but also the range of the zonule that is supported by the haptics and the location of the haptics within the zonule. In other words, there is little difference, in terms of IOL and zonule support, between an IOL with four haptics and one with two haptics if the haptics cannot support the zonule evenly. Since the zonule system has a circular configuration (360°) along the crystalline lens, force imbalances can occur with any number of haptics, unless the haptics are evenly positioned to support the whole zonule. In the case of the previously reported four-haptic IOLs (Akreos MI-60, BAUSCH+LOMB), even though there are four haptics, the interval between the haptics is larger in the long axis of the IOL, and the overall shape of the IOL is a rectangle with a long and short axis [27]. Thus, even if an IOL has four haptics, it may still provide less zonule support than an IOL with two haptics at a 180° interval, because the haptics are not evenly distributed in the zonule but only located on the long axis of the IOL. However, in the present study, IOL B (Microflex)—the four-loop haptic IOL—had four haptics, with an angle of around 90° between them (96° and 84°), suggesting that four haptics can support a circular zonule uniformly in 360°.

This is more pronounced when compared to the IOL A (2-loop plate haptic IOL). The two-loop plate haptic IOL had maintained a good anterior capsule opening 1 week after surgery, but this had decreased by 17.06% ± 15.99% after 2 months. In contrast, the four-loop plate haptic IOL only showed a 2.87% ± 6.03% decrease between 1 week and 2 months after surgery. In the comparison of the retro-illumination images (Fig. 1), the four-loop plate haptic IOL (b-1, b-2, b-3) showed a relatively circular anterior capsule opening after surgery, whereas the openings in the two-loop plate haptic IOL (a-1, a-2, a-3) and the two-plate haptic IOL (c-1, c-2, c-3) groups seemed to become elliptical over time. In particular, capsulorhexis margins appeared relatively stable where haptics were located. Where there were no haptics, the opening seemed to change to a narrower elliptical shape. Thus, it may be that the haptic does not support the zonule evenly and induces ACC. Furthermore, the IOL B group with the least ACC showed minimal change in SE value after surgery, suggesting that the haptic support also affects the position of the capsular bag, as well as the effective lens position (ELP).

Most commercially available IOLs are manufactured to be foldable within an injector to allow micro-incision cataract surgery (MICS). For this reason, IOLs are rectangular, and haptics are positioned at both ends of the long axis so that the IOL can be made smaller when folded. However, due to its shape, the four-loop plate haptic IOL used in the present study cannot be inserted into a 2.2-mm injector. Thus, an incision diameter of 2.4 mm was required to allow insertion. Although this may increase astigmatism after surgery, it may also decrease the risk of ACC and should be considered.

The four-loop plate haptic IOL maintained the anterior capsule opening well. However, the PCO generation was much more severe than in the other IOL groups. The Microflex—the four-loop plate haptic IOL used in the study—has a round-edged optic design, while the other IOLs have sharp-edged optic designs; this design is likely the reason for the severe PCO. Furthermore, the four-loop plate haptic of IOL B confers a large contact area with the capsule. This may allow space for active proliferation of LECs.

ACC can induce IOL decentration or tilting, and even IOL dislocation, whereas PCO, which is more common, can easily be treated using Nd:YAG laser posterior capsulotomy. Anterior capsule opening contraction can also be treated with Nd:YAG laser, and, more recently, femtosecond lasers have been used to enlarge a contracted capsule [29, 30]. Nonetheless, in severe cases, IOL tilting may not be improved after enlargement of the anterior capsule opening. Thus, it is important that surgeons prevent ACC as much as possible during surgery. Although many previous investigations have addressed ACC in Asian patients, the present study may be of significance as it is the first published study to focus on the location of IOL haptics and capsular bag stability. Many of the IOLs currently used may not provide an even force to support the zonule, because the haptics are not located at equal intervals around the haptics.

This study had several limitations. Firstly, the IOLs we compared differed in other characteristics besides haptic number and position. In particular, IOL B had a round optic edge that differed from those of the other IOLs. Therefore, it is problematic simply to compare the PCO among the three IOLs. Nonetheless, the present study may open a new line of inquiry based on the observation of previous studies that the optic edge does not affect the ACC [15, 16]. Secondly, although all known additional risk factors for ACC were excluded from the present study, there may be intrinsic factors other than IOL haptics that affect ACC. Thirdly, the ORGC size differed in the IOL A group (5.2 mm—0.2 mm smaller than in the IOL B and IOL C groups); this might have affected ACC. However, a previous paper reported that CCC size did not have a significant effect on capsule contraction. [31] Furthermore, significant ACC difference has been observed between groups with a CCC size of < 4.5 mm and those with a CCC size of 4.6–6.0 mm. [31] Based on previous reports, an ORGC difference of 0.2 mm would not have a significant effect on ACC.

In conclusion, the present study demonstrated that contraction of the anterior capsule opening was much smaller with the four-loop plate haptic IOL than with the two-loop plate haptic and two-plate haptic IOLs. ACC and IOL decentration vary depending on the location of the haptics within the capsular bag. Based on these results, when zonular weakness is detected during surgery, or when a condition that increases zonular instability is detected in the pre-operative examination, clinicians should consider selecting a four-plate haptic IOL that can support the zonule evenly.

Further studies, both clinical and histological, will be necessary to further elucidate the role of haptic position in ACC. It will also be necessary to develop a new IOL design that has evenly distributed haptics positioned within the capsular bag so that the rates of ACC and PCO can be lowered.

## Conclusions

The number and position of haptics within the capsular bag may affect ACC. We assume that supporting the zonules evenly plays a role in anterior capsular stability.

## Abbreviations

ACC: Anterior capsule contraction
ACO: Anterior capsule opacification
CCC: Continuous curvilinear capsulorhexis
CDVA: Corrected distance visual acuity
I/A: Irrigation/aspiration
IOL: Intraocular lens
LECs: Residual lens epithelial cells
MICS: Micro-incision cataract surgery
Nd:YAG: Neodymium:yttrium-aluminum-garnet
ORGC: Open Ring-shaped Guide for continuous curvilinear capsulorhexis
OVD: Ophthalmic viscoelastic devices
PCO: Posterior capsule opacification
